# A Multimode 28 GHz CMOS Fully Differential Beamforming IC for Phased Array Transceivers

**DOI:** 10.3390/s23136124

**Published:** 2023-07-03

**Authors:** Ayush Bhatta, Jeongsoo Park, Donghyun Baek, Jeong-Geun Kim

**Affiliations:** 1 Department of Electronic Engineering, Kwangwoon University, Seoul 01897, Republic of Korea; ayush@kw.ac.kr; 2 Department of Information Technology and Electrical Engineering, ETH, 8092 Zurich, Switzerland; jepark@ethz.ch; 3 School of Electrical Engineering, Chung-Ang University, Seoul 06974, Republic of Korea; dhbaek@cau.ac.kr

**Keywords:** 28 GHz, CMOS, phased array antenna, multi-channel, beamforming, transceiver, fifth generation (5G), mm wave, phase and gain control, multimode

## Abstract

A 28 GHz fully differential eight-channel beamforming IC (BFIC) with multimode operations is implemented in 65 nm CMOS technology for use in phased array transceivers. The BFIC has an adjustable gain and phase control on each channel to achieve fine beam steering and beam pattern. The BFIC has eight differential beamforming channels each consisting of the two-stage bi-directional amplifier with a precise gain control circuit, a six-bit phase shifter, a three-bit digital step attenuator, and a tuning bit for amplitude and phase variation compensation. The Tx and Rx mode overall gains of the differential eight-channel BFIC are around 11 dB and 9 dB, respectively, at 27.0–29.5 GHz. The return losses of the Tx mode and Rx mode are >10 dB at 27.0–29.5 GHz. The maximum phase of 354° with a phase resolution of 5.6° and the maximum attenuation of 31 dB, including the gain control bits with an attenuation resolution of 1 dB, is achieved at 27.0–29.5 GHz. The root mean square (RMS) phase and amplitude errors are <3.2° and <0.6 dB at 27.0–29.5 GHz, respectively. The chip size is 3.0 × 3.5 mm^2^, including pads, and Tx mode current consumption is 580 mA at 2.5 V supply voltage.

## 1. Introduction

The increasing demand for data communication brings about the emergence of 5G communications using mm-wave technology. Mm-wave phased array transceivers support multiple users operating with high data rates using broadband directional links between the mm-wave base stations and mobile devices [1,2,3,4,5,6,7]. To overcome the high free-space path losses at mm-wave frequencies, a phased array antenna has become one of the key techniques for mm-wave 5G communications. Therefore, RF transceivers must use a phased array design incorporating beamforming and beam-steering capabilities. However, realizing a compact and low-cost phased array architecture is a major concern [8,9,10]. Currently, to satisfy the demands for high-output power and low-noise performance, the front-end modules (FEM), power amplifiers, and low-noise amplifiers are designed using GaAs or GaN. However, other sub-blocks, such as driving amplifier, variable gain amplifier, phase shifter, attenuator, and power divider, are implemented using a CMOS due to its low cost, high integration, and efficient production capabilities. This results in the number of elements and system size increase. Consequently, the BFIC is realized with the CMOS technology to achieve a balance between performance and system compactness. In this work, a multimode BFIC is presented, which has the capability to operate in multimode within a single IC. The BFIC can support a number of antennas allowing wide flexibility in optimization and improving the beam steering, beam pattern, and SNR. In order to make the compact chip size and to improve the performance of phased array transceivers, multiple channels are integrated into a single chip. As the number of channels are integrated in the single BFIC, a fully differential structure is implemented to improve the isolation at each multi-channel port as well as to reduce the coupling effects.

In this paper, a multimode 28 GHz CMOS fully differential eight-channel BFIC is presented for a phased array transceiver. The BFIC incorporates a differential SPDT switch, which enables multimode operations. It has the capability to operate in 8Tx and 8Rx modes separately as well as in a combined 8Tx8Rx TDD mode. Furthermore, it is capable of operating in the four-channel mode, allowing the independent operation of 4Tx and 4Rx within a single BFIC. This flexibility in operation modes offers a range of choices depending on the specific requirements of the number of antennas. The eight-channel mode can integrate the number of antennas, resulting in a narrow beam, high EIRP, and improvement in the SNR suitable for mm-wave base stations. Furthermore, the ability of the BFIC to support a four-channel mode enhances its suitability for mobile devices by providing a compact and power-efficient chip solution.

## 2. Design of 28 GHz Eight-Channel Multimode Beamforming IC

**Figure 1:**
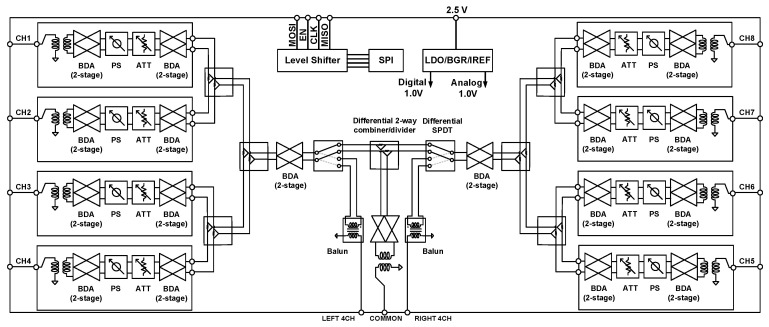
Block diagram of 28 GHz eight-channel multimode beamforming IC.

The RF-phase-shifting beamforming architecture has been adopted considering the power consumption, chip size, and design complexity. The block diagram of the 28 GHz eight-channel CMOS fully differential multimode BFIC is shown in Figure 1. All the circuits in the chip have a differential structure. A differential design reduces coupling between the channels and improves isolation. A simple and high-performance unit channel structure is used. Each channel consists of a differential two-stage bi-directional amplifier, a six-bit phase shifter, and a three-bit digital step attenuator. The multiple channel Tx/Rx signals are split or combined using a two-way power divider. The fully differential SPDT switch is implemented to control the multimode operations. The bi-directional amplifiers were distributed to deliver enough power to each channel. A Marchand balun is implemented in the common signal path of the four-channel for transforming single-ended and differential signals. To realize the compact phased array antenna system, the bi-directional architecture is employed. The area reduction is realized by sharing the passive phase shifters, attenuators, and power divider between the TX mode and the RX mode. 

**Figure 2:**
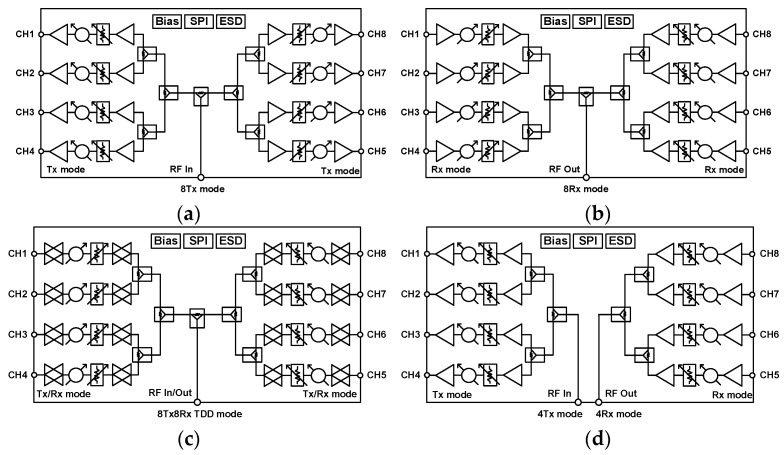
Simplified block diagram of multimode operation of 28 GHz eight-channel CMOS beamforming IC: (**a**) 8Tx mode, (**b**) 8Rx mode, (**c**) 8Tx8Rx TDD mode, and (**d**) 4Tx and 4Rx independent mode.

The 28 GHz eight-channel BFIC has the capability to operate in multimode, including the 8Tx and 8Rx modes separately, the 8Tx8Rx TDD mode, and also the 4Tx and 4Rx mode independently. When the SPDT switches are in the default state (off state) and all the eight-channel are set to Tx mode, it operates in 8Tx mode, as shown in Figure 2a. When all the eight-channel are set to Rx mode, then it operates as the 8Rx mode, as in Figure 2b. Additionally, by controlling the Tx/Rx mode select switches presented in each channel, it can be operated bi-directionally, which enables BFIC to be operated in the 8Tx8Rx TDD mode, as shown in Figure 2c. The BFIC is also able to operate in the four-channel mode. When the SPDT switches are on, the right side, the four-channel can be made to work in Tx mode and the left side four-channel can be made to work in Rx mode, as shown in Figure 2d. Hence, the BFIC can operate in the 4Tx and 4Rx mode independently. The bias, phase, and amplitude controls for each channel are digitally controlled by the SPI. Furthermore, the bandgap voltage reference (BGR), low drop output (LDO) regulator, and electrostatic discharge (ESD) protection circuits are integrated into the eight-channel BFIC to provide a stable DC bias.

### 2.1. Differential Two-Stage Bi-Directional Amplifier

**Figure 3:**
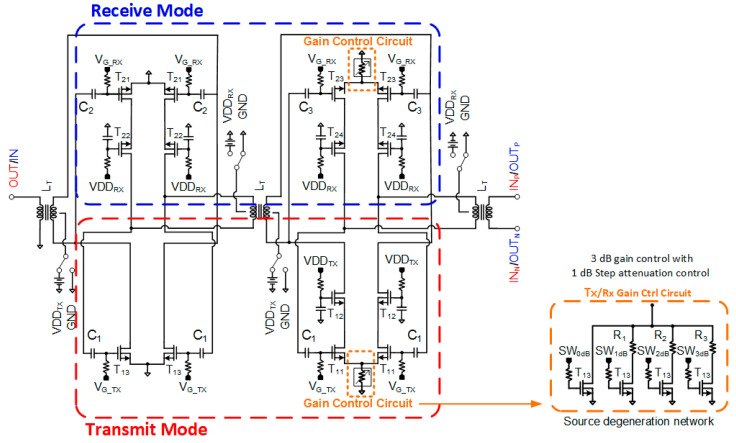
Schematic of the transformer coupled differential two-stage bi-directional amplifier.

The differential amplifier is designed with a transformer-coupled structure, which has the advantage of not requiring a DC-blocking capacitor and RF choke and also improves the bandwidth [11]. Figure 3 shows the circuit schematic of the transformer coupled differential two-stage bi-directional amplifier. The two-stage bi-directional amplifier uses transformers for input, inter-, and output stage matching. The Tx/Rx mode select switches are used to control the Tx and Rx mode at each channel. For the Tx mode operation, transistors, T_11_, T_12_, T_13,_ are turned on, while the Rx mode transistors, T_21_, T_22_, T_23_, T_24,_ are turned off and vice-versa in the Rx mode operation. Both the Tx mode and the Rx mode gain stages are designed with cascode amplifier topology with the source degenerative resistive circuit for precise gain control. A gain control of 3 dB with 1 dB step attenuation is implemented. The gain control circuit consists of four RF NMOS transistors and the attenuation resistor. The resistances of 1 dB, 2 dB, and 3 dB attenuation are 4 Ω, 9 Ω, and 14 Ω, respectively. 

**Figure 4:**
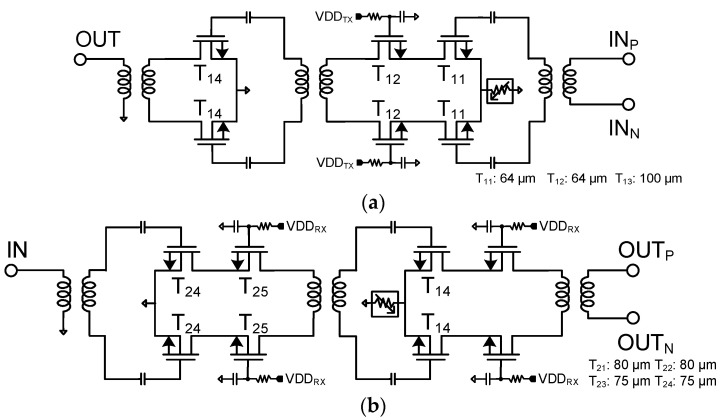
Equivalent circuit diagram of the differential two-stage bi-directional amplifier: (**a**) Tx mode and (**b**) Rx mode.

The equivalent circuit diagram of the Tx mode of the bi-directional amplifier is shown in Figure 4a. The Tx mode output stage is designed with common-source topology for high linearity. Figure 4b shows the Rx mode of the bi-directional amplifier. The Rx mode input stage is designed with cascode amplifier topology for high isolation between the input and the output ports. The proposed circuit configuration does not need additional SPDT switches for bi-directional operation, which reduces the chip area as well as insertion loss in the Tx and Rx modes. Furthermore, the inductors can be shared for both modes, resulting in the reduction of inductors. 

**Figure 5:**
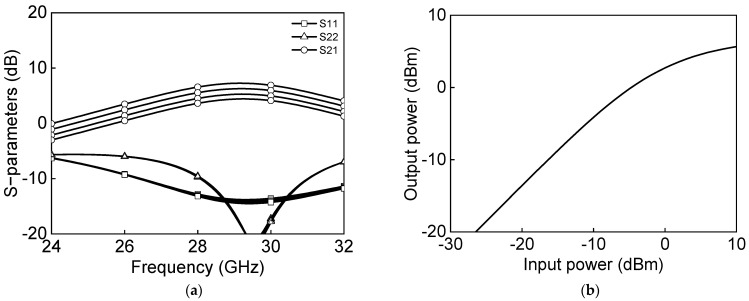
Simulated results of differential two−stage bi−directional amplifier in Tx−mode: (**a**) s−parameter (**b**) power characteristics.

**Figure 6:**
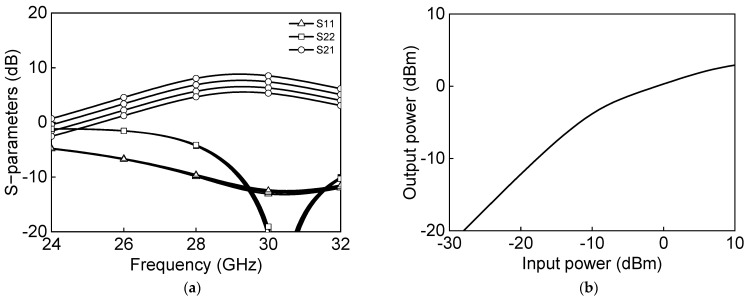
Simulated results of differential two−stage bi−directional amplifier in Rx−mode: (**a**) s−parameter and (**b**) power characteristics.

Figure 5a shows the simulated s-parameter results, and Figure 5b shows the power characteristics measurement of a differential two-stage bi-directional amplifier in the transmit mode. The transmitter-mode reference gain is 6.6 dB and output P1dB, and saturation power PSAT are −2.2 dBm and 5.6 dBm at 28 GHz. Figure 6a shows the simulated s-parameter results, and Figure 6b shows the power characteristics measurement of a differential two-stage bi-directional amplifier in the receive mode. The receiver-mode reference gain is 8.1 dB, and output P1dB and saturation power PSAT are −5.9 dBm and 2.8 dBm, respectively, at 28 GHz.

### 2.2. Differential Phase Shifters and Attenuators

**Figure 7:**
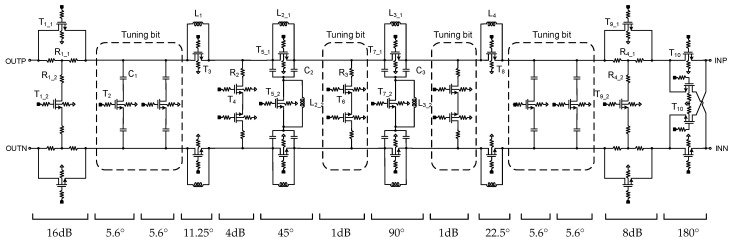
Schematic of the six-bit phase shifter and the three-bit digital step attenuator with tuning bit.

The schematics of the proposed six-bit differential phase shifter and three-bit digital step attenuator, including the tuning bit, are shown in Figure 7. The phase shifter and attenuator are designed with a differential structure since it has advantages, such as improved linearity and reduced coupling between the signals at different ports. The phase shifter and attenuator circuits are distributed in a single block to make the compact chip size. The phase shifter and attenuator bits are cascaded in such an order to obtain better input and output matching and lower insertion loss. 

The switched filter phase shifters are attractive for mm-wave design due to their high linearity and lack of DC power consumption. In addition, it can be implemented with a compact size and low insertion loss because it consists of lumped components, including NMOS transistor switches [12]. The six-bit differential phase shifter is designed with switched filter phase shifter structures. The 11.25° and 22.5° phase shifters are designed using a single inductor and bypass switch. When the series switch (T_3_ or T_8_) is on, the signal passes through the reference state. In the phase-shifting state, the switch (T_3_ or T_8_) is off and the signal passes through the series inductor (L_1_ or L_4_) with phase shifting of 11.25° and 22.5°, respectively. The 45° and 90° phase shifters are based on the switched-LC network using differential NMOS switches. In the phase-shifting state, T_5_1_ or T_7_1_ is off and T_5_2_ or T_7_2_ is on, making the π- network LPF. The signal is delayed when passing through the π- network LPF, making phase shifts of 45° and 90°, respectively. In the reference state, T_5_1_ or T_7_1_ is on and T_5_2_ or T_7_2_ is off, respectively. The inductors, L_2_2_ and L_3_2_, are adjusted to resonate with parasitic capacitance formed when transistors, T_5_2_ and T_7_2_, are off respectively. The design of the 180° phase shifter employs a minimalistic approach, relying solely on the cross-connected switch type to achieve the desired phase shift without the need for extra components, such as inductors or capacitors, resulting in a compact chip size. To achieve a tuning bit of 5.625°, a simple configuration consisting of a shunt NMOS transistor switch and a capacitor (C_1_) is used. To reduce the chip size, vertically stacked spiral inductors are used. All the inductors, MIM capacitors, and interconnection lines were designed using electromagnetic simulation. 

The attenuator is used in calibrating amplitude error as well as sidelobe level reduction in the radiation pattern of an antenna. For zero DC power consumption, the attenuator is designed in a passive type, which is beneficial to be used in a large-scale phased array antenna system. Various topologies are available for the design of attenuators, including the switched path type, the distributed type, and the switched Pi/T-type attenuator [13]. The switched Pi/T-type attenuator is chosen based on its small size and low insertion loss. This type of attenuator is designed to be compact and efficient, using only three resistors and NMOS transistor switches to achieve the desired attenuation. The switched T-type attenuator is determined by Equations 1 and 2 where Z_0_ is the transmission line characteristic impedance and A_dB_ is the desired attenuation in the dB scale.

(1) $$R_{1\;}{=Z}_{0}\left[\frac{10^{\frac{A_{\mathrm{dB}}}{20}}-1}{10^{\frac{A_{\mathrm{dB}}}{20}}+1}\right]$$

(2) $$R_{2}{=2Z}_{0}\left[\frac{10^{\frac{A_{\mathrm{dB}}}{20}}}{10^{\frac{A_{\mathrm{dB}}}{20}}-1}\right]$$

**Figure 8:**
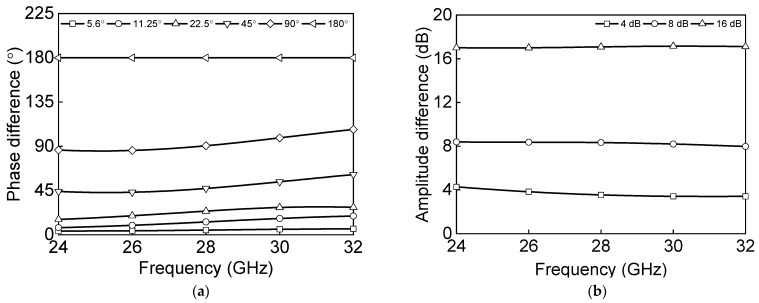
Simulated results: (**a**) phase difference of six-bit phase shifter and (**b**) amplitude difference of three-bit attenuator.

The differential 8 dB and 16 dB attenuators are designed using a switched T-type structure. The differential 4 dB attenuator and 1 dB attenuator are implemented with the shunt resistors, R_2_ and R_3_, and series switch transistors, T_4_ and T_6_, to reduce the chip size and loss. The attenuation state of 1 dB is used as a tuning bit to correct the amplitude error. Figure 8a shows the simulated phase difference of the main phase shift states of the six-bit phase shifter. Figure 8b shows the amplitude difference for each state of the three-bit attenuator.

### 2.3. Differential Two-Way Power Divider

**Figure 9:**
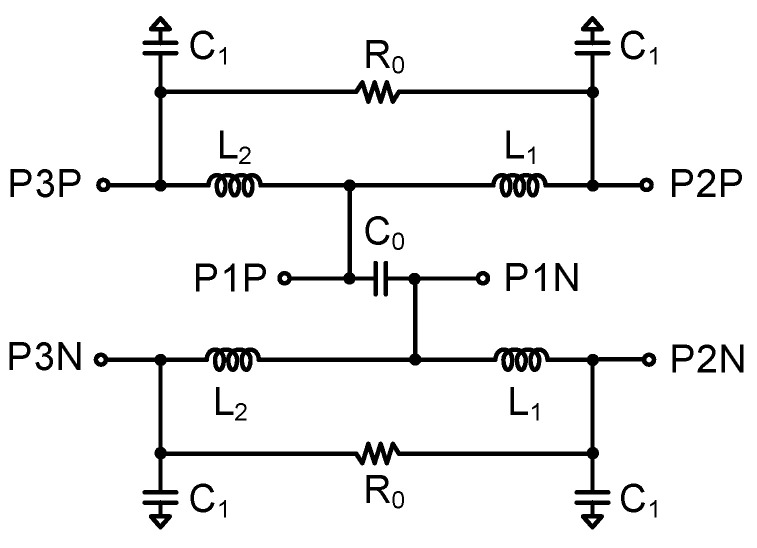
Schematic of the differential two-way power divider.

A power divider is implemented in the BFIC to split or combine the multiple channels transmit/receive signals. The two-way power divider is designed with differential structures, which is robust to parasitic inductances to the ground paths. The chip size is reduced using an artificial transmission line instead of the quarter-wavelength transmission line used in a conventional two-way power divider [14]. The schematic of the differential two-way power divider is shown in Figure 9. All capacitors in this circuit used metal-insulator-metal (MIM) capacitors.

(3) $$L=\frac{Z_{0}\sin\phi }{4\pi f},\;C=\frac{\tan\left(\frac{\phi }{2}\right)}{{2\pi \mathrm{fZ}}_{0}}$$

**Figure 10:**
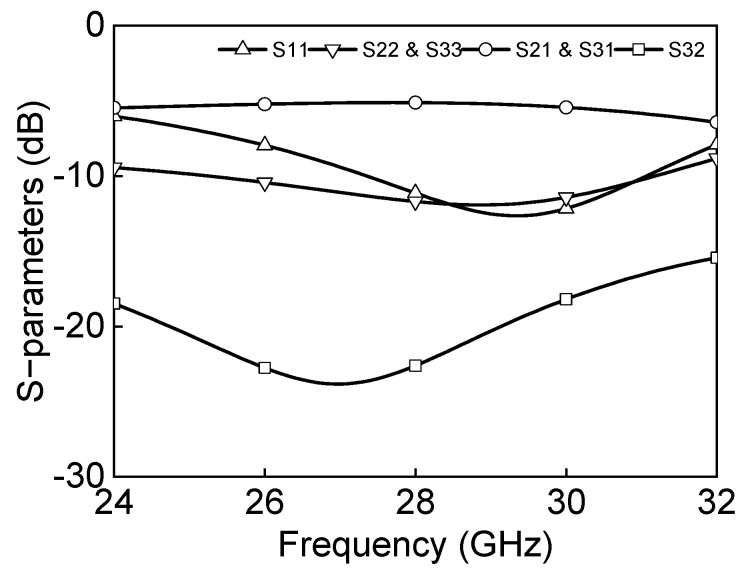
Simulation results of the differential two−way power divider.

The inductance and capacitance in a differential two-way power divider are determined by Equation (3) where Z_0_ is the transmission line characteristic impedance and *ϕ* is the phase of the transmission line. Figure 10 shows the simulated insertion losses, return losses, and isolation. The insertion losses are −5.1 dB, and input/output return losses are under 10 dB at 27–29.5 GHz. The isolation between P2P and P2N and P3P and P3N is under 20 dB at 25–29.5 GHz.

### 2.4. Marchand Balun

**Figure 11:**
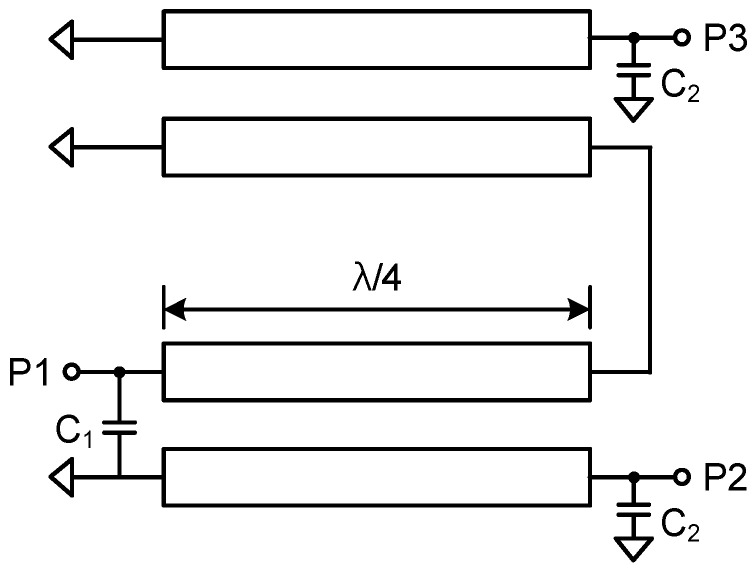
Schematic of the Marchand balun.

**Figure 12:**
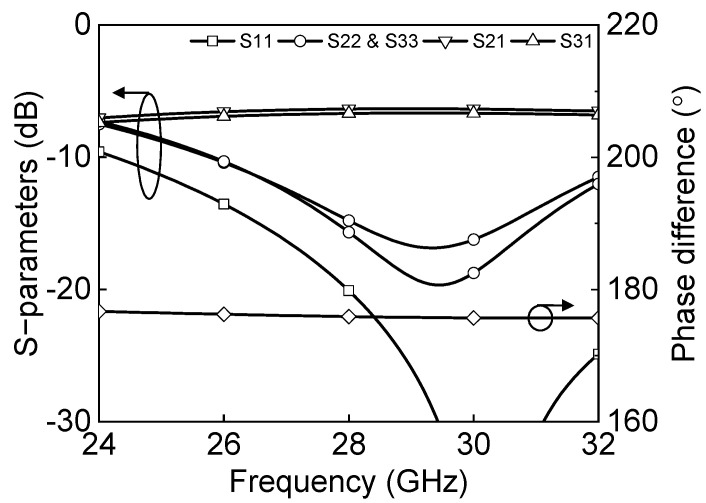
Simulation results of the Marchand balun.

The Marchand balun is implemented in transforming single-ended and differential signals. The Marchand balun is attractive due to its wideband performance, employing quarter wavelength coupled line sections. Figure 11 shows the schematic of the Marchand balun. The quarter-wavelength transmission lines are implemented as a vertically coupled structure, and the shunt capacitor helps compensate for impedance matching. Figure 12 shows the simulated insertion loss, return losses, and phase differences between differential output paths. The insertion losses of P1 to P2 and P1 to P3 are −6.3 dB and −6.7 dB at 27–29.5 GHz, respectively. The simulated return losses at each port are under 10 dB at 26–32 GHz, and the simulated phase difference is 176° at 26–32 GHz.

### 2.5. Differential SPDT Switch

**Figure 13:**
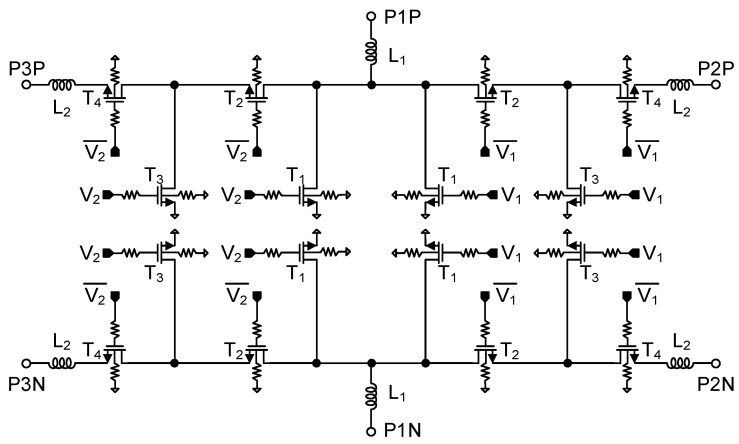
Schematic of the differential SPDT switch.

**Figure 14:**
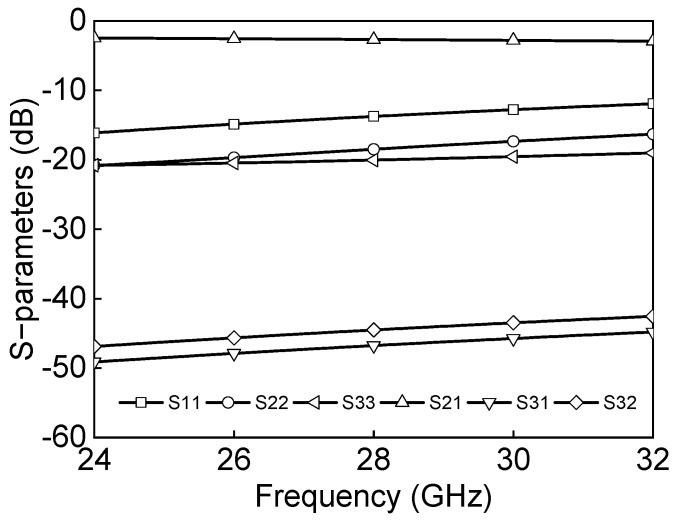
Simulation results of the differential SPDT switch.

A fully differential SPDT switch is designed to have high-power-handling capabilities for both the Tx and Rx paths and to have parasitic inductance robust performance as the CMOS switch structure has high insertion loss and low-power characteristics in high frequency. The body-floating technology is widely used in the CMOS switch to reduce insertion loss and improve power characteristics [15]. The body-floating technology can prevent leakage signals through the substrate and improve the insertion loss by adding a high-value resistor in the transistor body. Figure 13 shows the schematic of the proposed differential SPDT switch. The reflective SPDT switch is based on a series-shunt configuration for added isolation. The series transistors, T_2_ and T_4_, perform the main switching function, and the shunt transistors, T_1_ and T_3_, increase the isolation of the switch. In the design of SPDT switches, the gate terminals are biased through a large resistor to reduce the fluctuation in the VGD and VGS of the transistors due to the voltage swings at the drain and source terminals. Figure 14 shows the simulation results of the differential SPDT switch at output1 ON and output2 OFF states. The insertion loss of the differential SPDT switch is −2.7 dB, the return losses are under 10 dB, and the isolation is under 40 dB at 24–32 GHz. 

### 2.6. Serial Peripheral Interface

**Figure 15:**
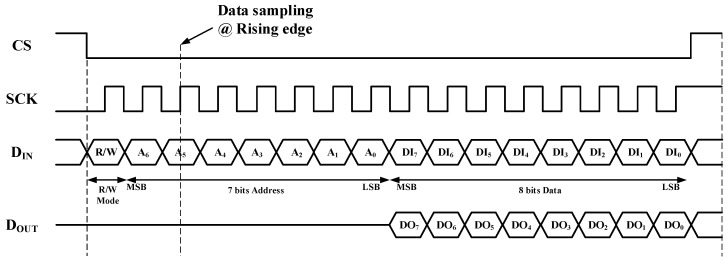
Timing diagram of the 16-bit SPI.

**Figure 16:**
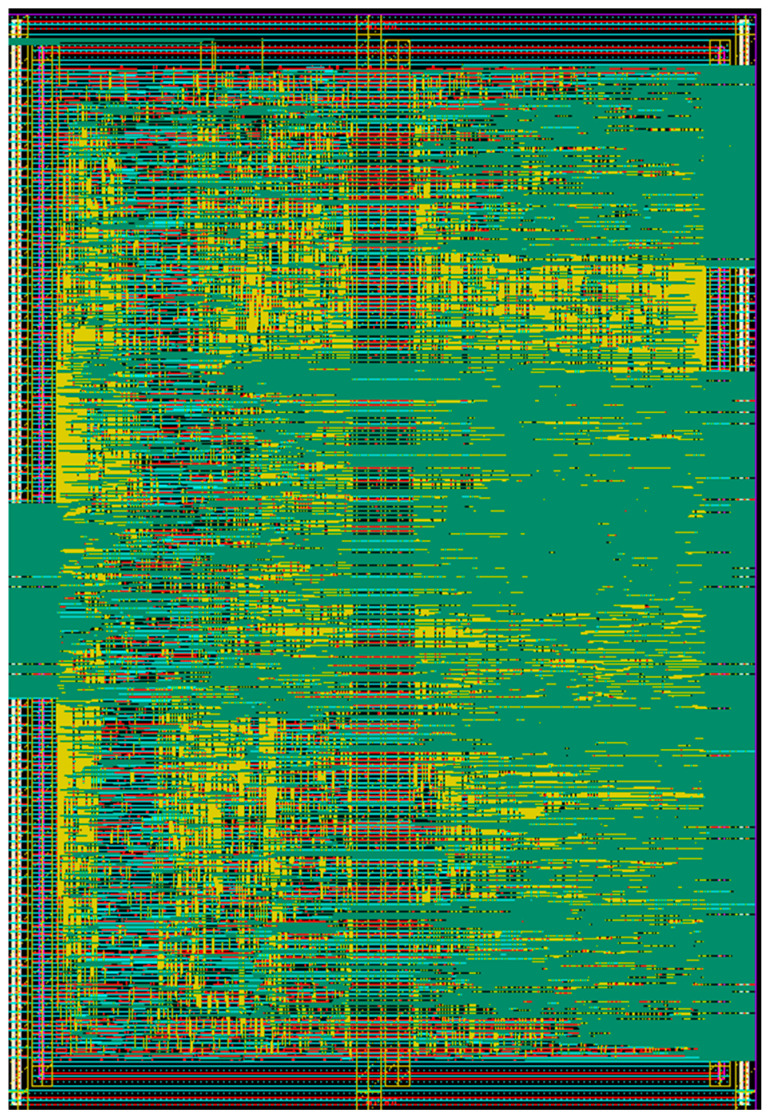
Layout of the synthesized SPI block.

The gain and phase control for each channel is digitally controlled by the SPI. The timing diagram of the 16-bit SPI is shown in Figure 15. The SPI configuration utilized SPI mode 0 with a CPOL (clock polarity) of 0 and a CPHA (clock phase) of 0 where the data is sampled on the clock’s rising edge and shifted on the falling edge. The SPI protocol for the BFIC involves a 16-bit register where the first bit represents the read/write mode, seven bits represent the address, and the subsequent eight bits represent the data. When executing a write operation, the first bit of the address byte is set as ‘0’. The desired seven-bit address is assigned to the address field, and the eight-bit data field is filled with the required control information for the phase shifter, attenuator, and bias operation. To perform the read operation, the first bit of the address byte is set as ‘0’. The SPI interface supports a maximum operating speed of 20 MHz. The SPI design has been synthesized using Verilog code. The layout of the synthesized SPI block is shown in Figure 16. The size of the synthesized SPI block is 0.30 × 0.44 mm^2^.

## 3. Measurement Results

**Figure 17:**
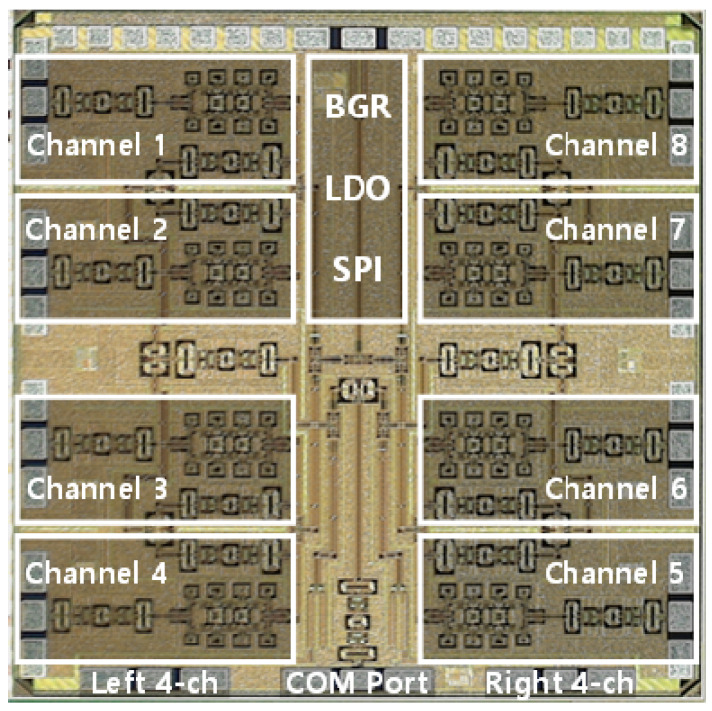
Microphotograph of the proposed 28 GHz CMOS eight-channel multimode beamforming IC.

The microphotograph of the fabricated 28 GHz eight-channel multimode BFIC fabricated in a commercial 65 nm CMOS technology is shown in Figure 17. The total area of the BFIC is 3.0 × 3.5 mm^2^, including pads. The DC current consumption of a 28 GHz eight-channel CMOS BFIC is 580 mA at 2.5 V supply voltage. The BFIC operates at a nominal voltage of 1.0 V and incorporates thin oxide technology. The measurements of the eight-channel BFIC are carried out with on-chip probing.

**Figure 18:**
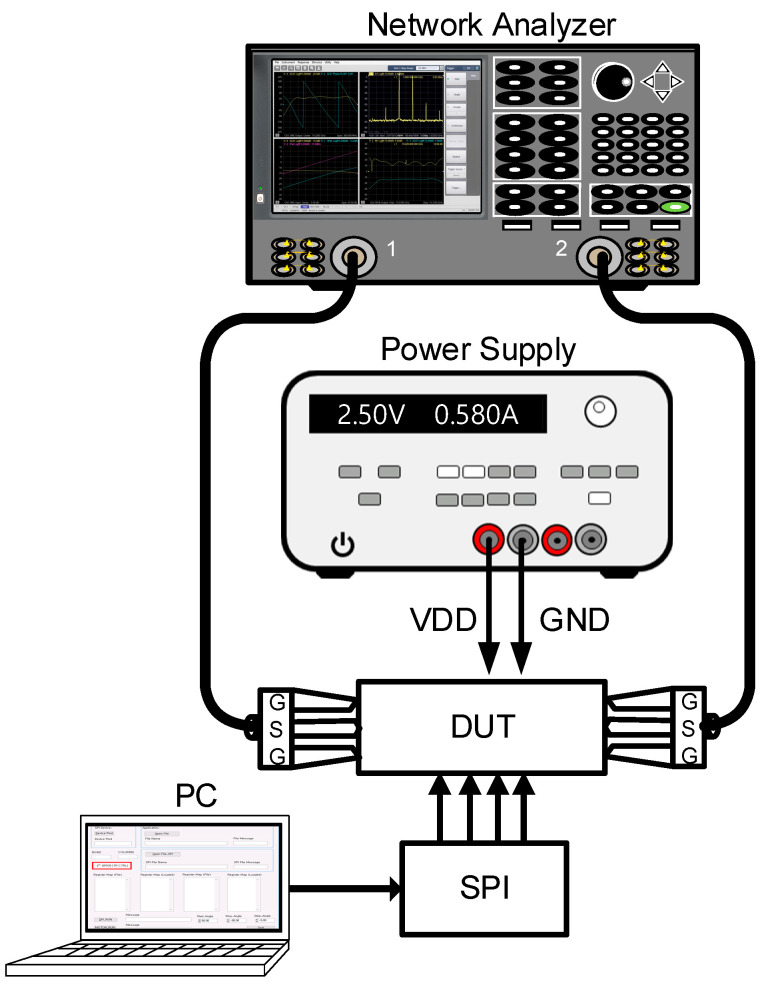
Measurement setup of on-wafer S-parameter of 28 GHz CMOS beamforming IC.

**Figure 19:**
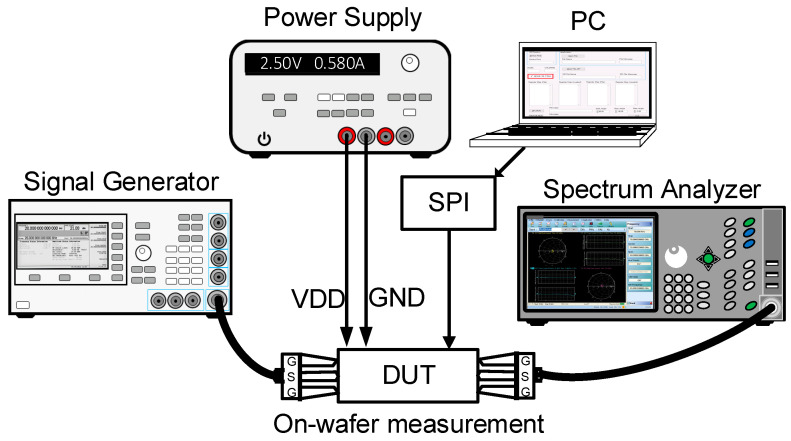
Measurement setup of on-wafer power characteristics of 28 GHz CMOS beamforming IC.

Figure 18 shows the on-wafer S-parameter measurement of the 28 GHz CMOS BFIC using a network analyzer. The short, open, load, and thru (SOLT) calibration was performed with the on-wafer probe station. The power characteristics are measured with the signal source and the spectrum analyzer. Figure 19 shows the on-wafer power characteristic measurement setup using the spectrum analyzer. This measurement method requires calibration from the measurement equipment port to the RF probe tip, including RF cables.

**Figure 20:**
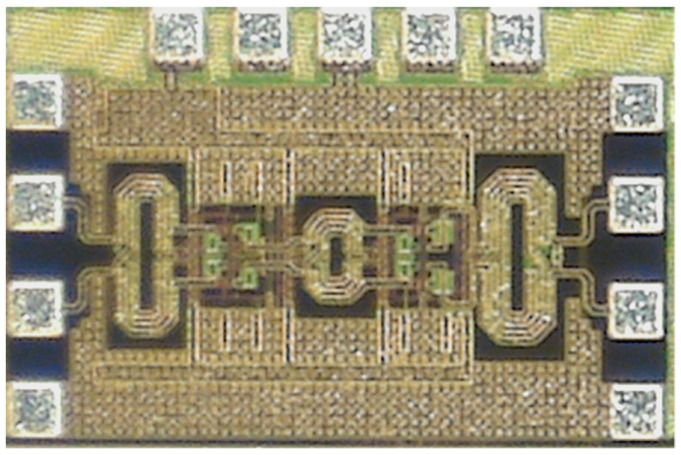
Microphotograph of the proposed differential two-stage bi-directional amplifier.

**Figure 21:**
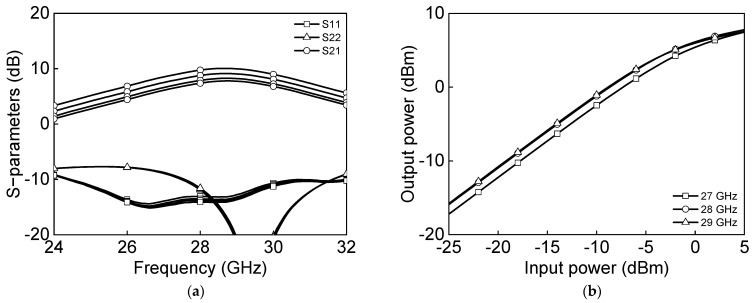
Measured Tx mode differential bi−directional amplifier: (**a**) S−parameters and (**b**) power characteristics.

**Figure 22:**
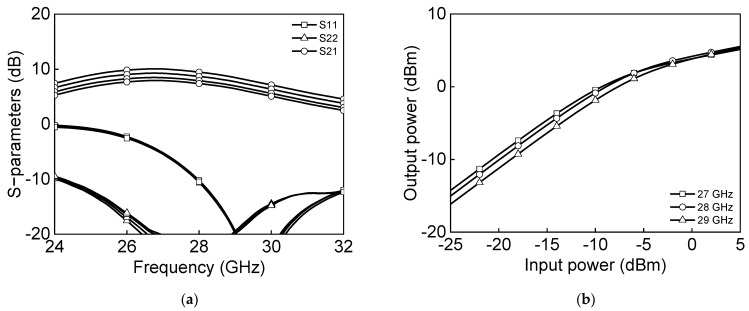
Measured Rx mode differential bi−directional amplifier: (**a**) S−parameters and (**b**) power characteristics.

**Figure 23:**
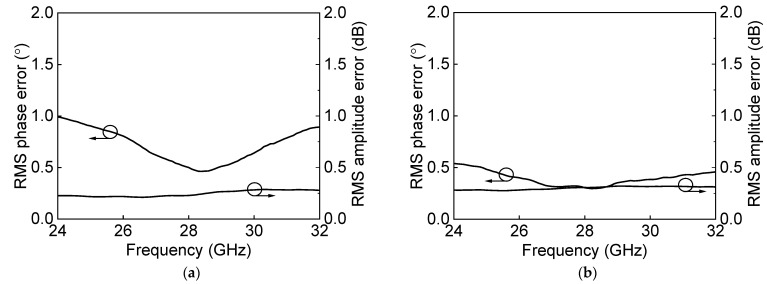
Measured RMS errors of the differential bi−directional amplifier with (**a**) Tx mode attenuation control and (**b**) Rx mode attenuation control.

Figure 20 shows the microphotograph of the fabricated 28 GHz differential two-stage bi-directional amplifier. The use of the transformer-coupled structure makes the compact chip size. The chip size of the differential two-stage bi-directional amplifier is 0.44 × 0.28 mm^2^ without pads. Figure 21a shows the measured S-parameter in the Tx mode of the bi-directional amplifier. The measured Tx mode gain is around 9 dB, and return losses are under 10 dB at 27.0–29.5 GHz. The gain control of 3 dB is achieved with an attenuation step of 1 dB. Figure 21b shows the power characteristics in the Tx mode of the bi-directional amplifier. The measured Tx mode outputs, P_1dB_ and P_SAT_, are 3.1 dBm and 7.8 dBm at 28 GHz, respectively. Figure 22a shows the measured S-parameter in the Rx mode of the bi-directional amplifier. The measured Rx mode gain is around 9 dB, and return losses are under 10 dB at 27.0–29.5 GHz. Due to the additional parasitic elements, the measured Rx mode gain operating frequency is slightly downshifted. Figure 22b shows the power characteristics in the Rx mode of the bi-directional amplifier. The measured Rx mode outputs, P_1dB_ and P_SAT_, are −0.1 dBm and 5.5 dBm at 28 GHz, respectively. Figure 23a shows the RMS phase error and RMS amplitude error in the Tx mode of the differential two-stage bi-directional amplifier with attenuation control of 3 dB with an attenuation step of 1 dB. The RMS phase error is <0.5° at 27.0–29.5 GHz, and the RMS amplitude error is <0.2 dB at 27.0–29.5 GHz. Figure 23b shows the RMS phase error and RMS amplitude error in the Rx mode of the differential two-stage bi-directional amplifier with attenuation control of 0 dB to 3 dB. The RMS phase error is <0.3° at 27.0–29.5 GHz, and the RMS amplitude error is <0.3 dB at 27.0–29.5 GHz.

**Figure 24:**
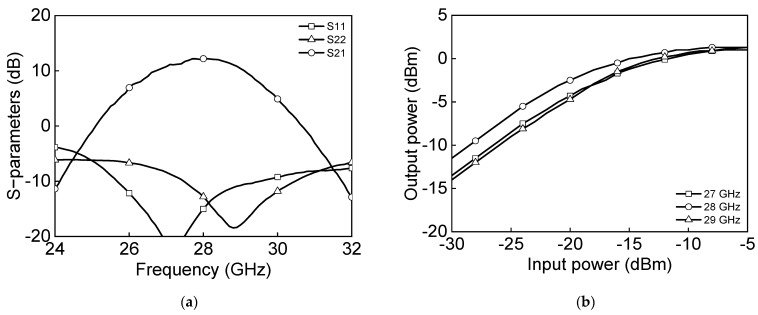
Measured Tx mode: (**a**) S−parameters and (**b**) power characteristics.

**Figure 25:**
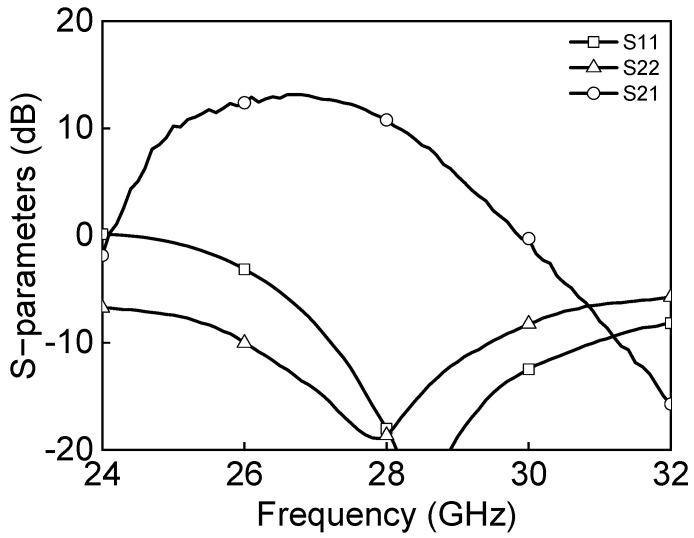
Measured Rx mode S−parameters.

**Figure 26:**
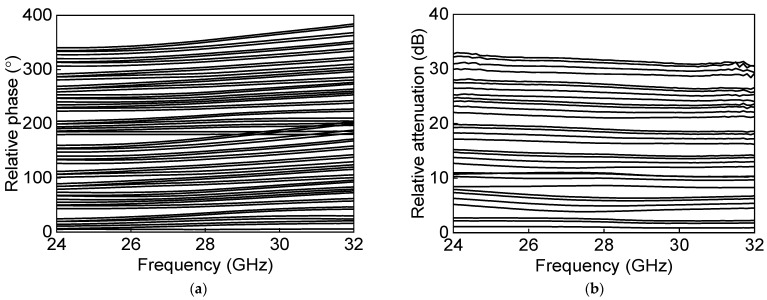
Measured (**a**) phase characteristics and (**b**) attenuation characteristics.

**Figure 27:**
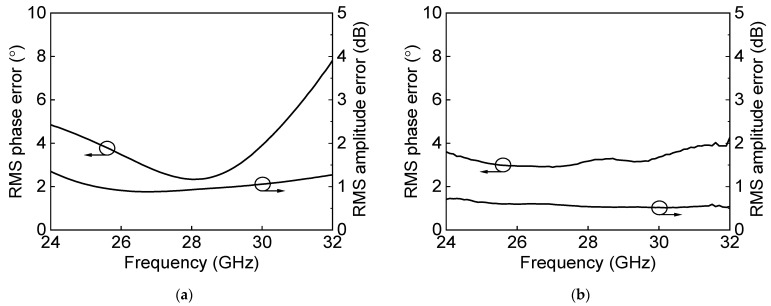
Measured RMS errors with (**a**) phase control and (**b**) attenuation control.

Figure 24a shows the measured S-parameter results in the Tx mode of the eight-channel BFIC. The measured Tx mode gain is around 11 dB, and return losses are under 10 dB at 27.0–29.5 GHz. Figure 24b shows the measured power characteristics in the Tx mode. The measured Tx mode outputs, P_1dB_ and P_SAT_, are −2.5 dBm and 1.3 dBm at 28 GHz, respectively. Figure 25 shows the measured S-parameter results in the Rx mode of the eight-channel BFIC. The measured Rx mode gain is around 9 dB, and return losses are under 10 dB at 27.0–29.5 GHz. The operating frequency of the measured Rx mode gain is slightly downshifted due to the presence of additional parasitic elements. Figure 26a shows the measured relative phase characteristics in all the phase states. A phase variation range of 0° to 354° with a phase resolution of 5.625° is achieved. Figure 26b shows the measured attenuation characteristics in all the attenuation states. The attenuation range of 31 dB, including the gain control bits with an attenuation resolution of 1 dB, is achieved. To quantitively check the phase-shifting performance of the BFIC, the RMS phase and amplitude errors in all the phase shift states are presented. Figure 27a shows the measured RMS phase error and amplitude error when varying the phase states. The measured RMS phase error is <3°, and the RMS amplitude error is <2 dB at 27.0–29.5 GHz. Figure 27b shows the measured RMS phase error of <3.2° and the RMS attenuation error of <1 dB at 27.0–29.5 GHz when varying the attenuation states. 

Table 1 summarizes the performance of this work and compares it with previously published 28 GHz beamforming ICs. The 28 GHz eight-channel multimode BFIC is designed with a differential structure to reduce interference from adjacent channels. The differential structure also allows common-mode rejection and less coupled signal at each port for multi-channel. The proposed BFIC has comparable RMS phase and amplitude error than the same RF phase-shifting architecture. The proposed BFIC allows for multimode operation based on the number of antenna requirements. The single BFIC is capable of operating in 8Tx and 8Rx modes separately as well as in a combined 8Tx8Rx TDD mode. Furthermore, it is capable of operating in four-channel mode, allowing the independent operation of 4Tx and 4Rx within a single BFIC.

## 4. Conclusions

This paper presents a multimode 28 GHz fully differential eight-channel BFIC in 65 nm CMOS technology for phased array transceivers. The bi-directional amplifier is implemented with a transformer coupled structure for compactness and low insertion loss. The cross connected quad switch type 180° phase bit and a differential two-way power divider using artificial transmission lines make the design compact and lower power consumption. The chip size is 3.0 × 3.5 mm^2^, including pads. The BFIC is capable of operating in multiple modes, enabling it to operate in the 8Tx and 8Rx modes separately, the 8Tx8Rx TDD mode, and also in four-channel mode 4Tx and 4Rx independently. The proposed 28 GHz fully differential eight-channel BFIC with a multimode operation can be implemented in mm-wave base stations and mobile devices.

## Figures and Tables

**Table 1:** Summary of 28 GHz multimode beamforming IC for phased array transceiver.

Ref.	This Work	[4]	[16]	[17]
Tech (CMOS)	65 nm	65 nm	65 nm	28 nm
Freq. (GHz)	27.0–29.5	26.5–30	26.5–29.5	25.8–28
Number of channels	8 TRx	4TRx	4 TRx	8 TRx
Single-ended/Differential	Differential	Differential	Differential	Single-ended
Tx/Rx Gain (dB)	11/9	18.6/14.8	9/11	N/A
P_1dB_/P_SAT_ (dBm)	−2.5/1.3	13.3/-	15.7/18	9.5/10.5
Phase shift step (^o^)	6 bit/5.6	6 bit/5.6	2 + 3 + 10 bit/0.3 **	3 bit/45
RMS amplitude error (dB)	0.6	0.21	0.04	1
RMS phase error (^o^)	3.2	1.4	0.3	7
Tx/Rx PDC of 1channel (mW)	181/181	73/-	299/148	85/50 *
Mode	8Tx only 8Rx only 8Tx8Rx TDD 4Tx4Rx- independent	4Tx only 4Rx only 4Tx4Rx- independent	4Tx only 4Rx only 4Tx4Rx- independent	8Tx only 8Rx only 8Tx8Rx TDD
Chip Size (mm^2^)	10.5	0.92 ***	12	7.3

* Estimated from the data. ** Step limited by phase error. *** One-Channel core size.

## Data Availability

The data can be obtained from the authors on request.
